# Design of a randomized controlled trial of physical training and cancer (Phys-Can) – the impact of exercise intensity on cancer related fatigue, quality of life and disease outcome

**DOI:** 10.1186/s12885-017-3197-5

**Published:** 2017-03-27

**Authors:** Sveinung Berntsen, Neil K Aaronson, Laurien Buffart, Sussanne Börjeson, Ingrid Demmelmaier, Maria Hellbom, Pernille Hojman, Helena Igelström, Birgitta Johansson, Ronnie Pingel, Truls Raastad, Galina Velikova, Pernilla Åsenlöf, Karin Nordin

**Affiliations:** 1 Dept. of Public Health and Caring Sciences, Lifestyle and rehabilitation in long term illness, Uppsala University, Box 564, 75122 Uppsala, Sweden; 2 Dept. of Public Health, Sport and Nutrition, University of Agder, Gimlemoen 25, 4604 Kristiansand, Norway; 3 Division of Psychosocial Research & Epidemiology, The Netherlands Cancer Institute, Plesmanlaan 121, 1066CX, Amsterdam, The Netherlands; 4 Departments of Epidemiology & Biostatistics and Medical Oncology, VU University Medical Center, PO Box 7057, 7007 MB Amsterdam, the Netherlands; 5 Dept. of Medical and Health Sciences, Division of Nursing Science, Linköping University Campus Valla, 581 83 Linköping, Sweden; 6 Division of Oncology and Pathology, Dept. of Clinical Sciences, Lund University, Box 117, 221 00 Lund, Sweden; 7 Centre of Inflammation and Metabolism, Copenhagen University Hospital, Blegdamsvej 9, 2100 Copenhagen, Denmark; 8 Experimental and Clinical Oncology, Dept. of Immunology, Genetics and Pathology, Uppsala University, Box 564, 75122 Uppsala, Sweden; 9 Dept. of Physical Performance, Norwegian School of Sport Science, Sognsveien 220, 0863 Oslo, Norway; 10 Leeds Institute of Cancer and Pathology, St James’s University Hospital LEEDS LS9 7TF University of Leeds, Leeds, UK; 11 Dept. of Neuro Science, Physiotherapy, Uppsala University, Box 564, 75122 Uppsala, Sweden

**Keywords:** Cancer, Physical exercise, Behaviour change techniques, Fatigue, Biological mechanism, Quality of life, Randomized controlled trial

## Abstract

**Background:**

Cancer-related fatigue is a common problem in persons with cancer, influencing health-related quality of life and causing a considerable challenge to society. Current evidence supports the beneficial effects of physical exercise in reducing fatigue, but the results across studies are not consistent, especially in terms of exercise intensity. It is also unclear whether use of behaviour change techniques can further increase exercise adherence and maintain physical activity behaviour. This study will investigate whether exercise intensity affects fatigue and health related quality of life in persons undergoing adjuvant cancer treatment. In addition, to examine effects of exercise intensity on mood disturbance, adherence to oncological treatment, adverse effects from treatment, activities of daily living after treatment completion and return to work, and behaviour change techniques effect on exercise adherence. We will also investigate whether exercise intensity influences inflammatory markers and cytokines, and whether gene expressions following training serve as mediators for the effects of exercise on fatigue and health related quality of life.

**Methods/design:**

Six hundred newly diagnosed persons with breast, colorectal or prostate cancer undergoing adjuvant therapy will be randomized in a 2 × 2 factorial design to following conditions; A) individually tailored low-to-moderate intensity exercise with or without behaviour change techniques or B) individually tailored high intensity exercise with or without behaviour change techniques. The training consists of both resistance and endurance exercise sessions under the guidance of trained coaches. The primary outcomes, fatigue and health related quality of life, are measured by self-reports. Secondary outcomes include fitness, mood disturbance, adherence to the cancer treatment, adverse effects, return to activities of daily living after completed treatment, return to work as well as inflammatory markers, cytokines and gene expression.

**Discussion:**

The study will contribute to our understanding of the value of exercise and exercise intensity in reducing fatigue and improving health related quality of life and, potentially, clinical outcomes. The value of behaviour change techniques in terms of adherence to and maintenance of physical exercise behaviour in persons with cancer will be evaluated.

**Trial registration:**

NCT02473003, October, 2014.

## Background

Cancer-related fatigue (CRF) is a multidimensional concept including physical, social, emotional, psychological and biological components experienced by persons treated for cancer. The biological mechanisms underlying CRF are not well understood [1]. CRF is reported in up to 90% of persons with cancer during adjuvant treatment with radiation therapy, chemotherapy and/or endocrine therapies [2, 3]. Clinically relevant levels of CRF have further been reported in approximately one-third of cancer survivors up to 6 years post-treatment [3, 4]. CRF has serious impact on the person’s health-related quality of life (HRQoL) [5, 6] and causes a considerable challenge to society in humans and economic terms [7]. Analysis shows that cancer-related mortality costs €75 billion in Europe in 2008 due to loss of productive life [8].

Systematic reviews emphasize the potential physical and psychosocial benefits from rehabilitation programs including physical exercise [9, 10]. Previous studies have suggested that exercise interventions in persons with cancer may be cost-effective [11–13]. At the same time, there are significant challenges associated with a change of lifestyle during and following cancer treatment [14, 15]. Studies that have evaluated the effectiveness of exercise interventions on CRF are not consistent with respect to exercise volume or intensity to be recommended [16, 17], highlighting the need for more research. Despite the growing body of evidence that exercise is beneficial to persons both during and after oncological treatment, persons still avoid exercise following the cancer diagnosis and reduce their physical activity levels [18, 19]. More research is needed regarding how to enable individuals to become and stay physically active [20] and which techniques [21] to use in order to facilitate this behavioural change [22].

The results of a meta-analysis indicate that physical exercise has a significant, clinically relevant positive effect on CRF [9, 16, 17, 23]. However, the results of randomized controlled trials are not consistent, and it is unclear which exercise intensity level is most appropriate and most efficacious for the management of CRF. From a methodological perspective, not all studies have had CRF as their primary outcome, and some studies were underpowered to detect a clinically relevant effect of exercise [9, 16, 17, 23].

From a bio-psychosocial perspective, exercise may comprise many different behaviours influenced by physical, psychological and contextual determinants of which some are amenable to change and others are not. Previous research among cancer populations has identified modifiable psychological determinants of physical exercise; the most salient being readiness to change, self-efficacy for exercise and perceived behavioural control [24, 25]. BCTs can be used to facilitate and encourage exercise behaviour change. This includes exploring motivational issues (e.g., pros and cons of exercise, readiness to change and self-efficacy for physical exercise) and using self-regulatory strategies (e.g. individual goal-setting, self-monitoring and analysing one’s own physical exercise behaviours, and developing plans for maintenance of exercise behaviour including generalization to various settings) [21, 26].

Endurance exercise has been shown to increase the level of anti-inflammatory cytokines, leading to a systematic lowering of pro-inflammatory cytokine response as part of the training adaptation and indicating a general anti-inflammatory effect of exercise [27]. There is some evidence that this anti-inflammatory response also depends on exercise intensity [27]. However, limited knowledge exists in persons with cancer [28]. In addition to anti-inflammatory responses, muscular expression of apoptotic and metabolic markers may also be relevant to the interplay between physical exercise and fatigue [29, 30]. While the causes of CRF among cancer survivors are not yet fully understood, decreased fitness, as well as immune and cytokine dysregulation have been suggested to play a role [9, 27]. Accumulating evidence also suggests that several pathways, including chronic inflammation, autonomic imbalance, HPA-axis dysfunction, and/or mitochondrial damage, could contribute to the disruption of normal neuronal function and result in CRF [28].

To summarize, there is evidence supporting the beneficial effects of exercise during adjuvant treatment in cancer persons, but the findings with regard to fatigue are inconsistent. Also, the mechanisms through which exercise reduces or prevents CRF are still not fully understood. There is still a need for large-scale and well-designed studies including persons at high risk of developing CRF during treatment [31]. In addition, more research is needed to determine the optimal level, intensity, and type of exercise, as well as how tailored BCTs and structured exercise regimes may contribute to increased exercise adherence and maintenance of physical activity behaviour throughout the cancer survivorship period [10]. Recently Barsewick et al. [1] highlighted the need to conduct longitudinal research on the interrelated bio-behavioural mechanisms underlying CRF, and to test mechanistic hypotheses within the context of CRF intervention research. Few studies have investigated the effects of physical exercise on genetic biomarkers and systemic inflammatory markers in cancer persons with and without CRF [31]. The present study aims to address many of these issues.

The *main aim* of this randomized controlled trial, Phys-Can, is to determine the effects of low-to-moderate and high intensity physical exercise with or without BCTs on CRF and HRQoL in persons with cancer, both during treatment and in the long-term, post-treatment survivorship period. Additionally, the trial will investigate the role of inflammation, cytokines and gene expression in the development and maintenance of CRF, as well as the cost-effectiveness of physical exercise programs during cancer treatment.

More specific, our objectives are to:

1. Investigate the effects of low-to-moderate intensity exercise compared to high intensity exercise on person-reported outcomes (CRF as primary endpoint), chemotherapy/radiation completion rates, medical (oncology) adverse effects, physical activity and daily function, during adjuvant/curative treatment and at long-term-follow up. In addition, intervention effects on treatment tolerability and time to recurrence of cancer will be investigated
2. Investigate if supplemental BCTs increase adherence to and the efficacy of a physical exercise intervention during and after adjuvant therapy.
3. Explore the regulation of systematic inflammatory markers and muscular expression of cytokines in response to physical training and following training to investigate whether these serve as mediators for the effects on physical exercise on CRF and HRQoL.
4. Evaluate the cost-effectiveness of physical exercise interventions for CRF from a societal perspective.

## Methods

The Phys-Can project is a randomized controlled trial with a preceding descriptive observational study to be used for comparisons.

The purpose of the observational study is to monitor how disease and treatment are associated with the person’s cardiorespiratory fitness, mental well-being, quality of life and patterns of physical activity over time. The same inclusion/exclusion criteria as in the Phys-Can intervention study were used. Persons with breast, colorectal and prostate cancer about to begin their neoadjuvant and/or adjuvant therapy in Uppsala, Linköping and Malmö/Lund were asked to participate. Inclusion started 15th of September 2014 and was terminated 28th of February 2015. A total of 95 persons were included. They followed the same intervals in terms of physical testing and self-report questionnaires as the persons in the Phys-Can intervention study, but were not participating in any exercise intervention. In addition to physical tests and person-reported outcomes, data from medical records in adjunction to adjuvant treatment and register data were collected to be used for health economic analyses.

Directly after completion of enrolment of persons in the observational study (15th of March 2015), the inclusion to the randomized controlled trial was started. Persons recently diagnosed with breast cancer, colorectal cancer or prostate cancer, scheduled for neoadjuvant chemotherapy (breast cancer) or endocrine therapy (prostate cancer), and/or adjuvant chemotherapy (breast- and colorectal cancer), adjuvant radiotherapy (breast cancer) and/or adjuvant endocrine therapy (breast- and prostate cancer) or radiotherapy with curative intent without additional endocrine therapy (prostate cancer) are invited to participate. Consecutive persons are recruited from Uppsala, Lund/Malmö and Linköping University hospitals. Persons who are not able to perform basic activities of daily living, who show cognitive disorders or severe emotional instability, who suffer from other disabling co-morbid conditions that might contraindicate physical exercise (e.g. heart failure, chronic obstructive pulmonary disease orthopaedic conditions or neurological disorders) are ineligible. All persons are assessed by a cancer specialist (oncologist or surgeon) regarding contraindications for high intensity exercise. Persons without contraindications receive a brief information sheet about the Phys-Can study, and are informed that there are no medical contraindications for them to participate. Next, eligible persons are contacted by one of the research staff and provided with more detailed information, both written and verbal, and are given the opportunity to ask questions. Those who agree to participate in the trial provide written informed consent.

The Phys-Can study was approved by the Regional Ethical Review Board in Uppsala, Sweden (Dnr 2014/249) and registered in ClinicalTrials.gov (TRN = NCT02473003, Oct, 2014).

### Design

**Fig. 1:**
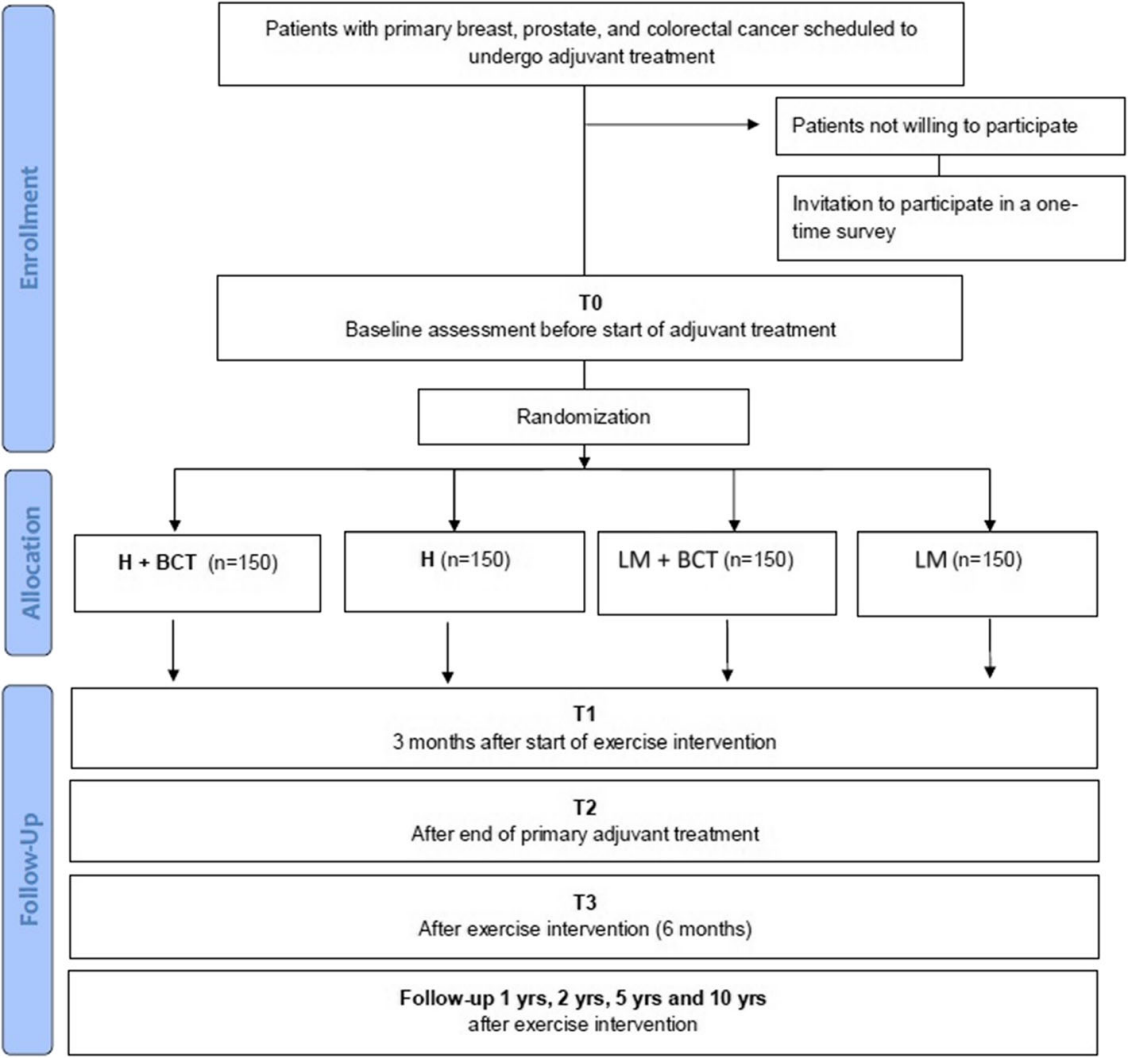
Diagram of the participant flow through enrolment, baseline measurement, randomization, and follow-up. H = high intensity training; L = low intensity training; BCT = Behaviour Change Technique

The design is a 2×2 factorial design where participants are randomized to one of the following four groups (Fig. 1):

1. individually tailored high intensity exercise with (H + BCTs) or
2. without BCTs (H)
3. individually tailored low-to-moderate intensity exercise with (LM + BCTs) or
4. without BCTs (LM).

The RCT is stratified and within each stratum randomization is carried out following a permuted block design with 8 participants per block. The defined strata are the three hospitals (Linköping, Lund and Uppsala) and three cancer sites yielding 9 strata. When all baseline measurements are finalized and reported in the project’s electronic case report form, the randomization is generated automatically in a web-portal (described below) of participants to an exercise group according to predefined strata (see above). The personnel in charge of recruitment then contact the participant with information on study condition and the first visit to the gym is scheduled.

All participants will exercise at least twice weekly for a period of 6 months under the guidance of trained coaches. Training intensity is 40–50% (LM) or 80–90% (H) of each individual’s heart rate reserve (Heart rate reserve = HRpeak-HRrest) and maximal muscle strength.

### Sample size

The calculated sample size was based on detecting the main factorial effects of each factor on Multidimensional Fatigue Inventory (MFI) Physical Fatigue (PF) after completed intervention [32–34]. The target main effect size was determined to be 2, which according to Purcell et al. [35] is the minimal clinically important difference for the MFI-PF. We assumed that the standard deviation (SD) of PF was 5 in all four treatment arms, which is the SD reported in Purcell et al. [35], Hagelin et al. [36] and in an ongoing study within our research group (manuscript in preparation). Because three tests (two main and one interaction effect) were planned, the 5% significance level was adjusted using Bonferroni correction. In order to have 80% power to detect a main factorial effect of 2 under the null hypothesis of no effect, 67 individuals in each of the four treatment arms are needed, with a total sample size of 268 individuals.

Further adjustments of the sample size were necessary for several reasons. First, the sample size calculation was based on the assumption of no interaction effects. However, Montgomery et al. [29] argue that, if anticipating quantitative interactions, the trial should be powered to detect those effects. Interaction effects often are much smaller in size compared to the main effects. In clinical trials, it is therefore rare to have 80% power to detect the interaction effect. For instance, given the calculated sample size in the present study, the trial would have a power of only 22% to detect an interaction effect of 1. However, in the present study a possible interaction between the factors is of clinical interest. Thus, the original sample size was doubled, increasing the power to detect the interaction between to approximately 50%. Second, to account for missing data and drop-outs, the original sample size was increased further by 30%. Third, given that we include a baseline assessment, and assuming a moderate correlation (0.3) between the MFI-PF at baseline and follow-up assessments, the sample size can be reduced by approximately 10% [37]. Combining the above, we concluded that including approximately 150 individuals in each treatment arm, 600 individuals in total, should meet the statistical requirements of the trial.

### Description of interventions

All participants are offered guided exercise for 6 months. This is equal to the most extensive adjuvant treatment period with the exception for endocrine therapy which may continue up to 10 years (for persons with breast cancer). It is also an appropriate period of time to achieve physical exercise effects and to establish a physically active lifestyle [22, 38]. Trained coaches will guide both resistance and endurance exercise.

### Resistance training

The resistance training will be performed at public gyms during two supervised sessions per week. During the first six weeks after inclusion the participants become familiar with the exercises and tests as well as how to use the Omni-scale for self-reported perceived exertion [39] included in the resistance program. During these six weeks, there will be a progression in sets and power output as well as a reduction in the number of repetitions, resulting in each participant having an individualized program. The following exercises are included in the program and performed on machines; seated leg press, chest press, leg extension, seated row, seated leg curl, and seated overhead press using dumbbells. In addition, participants are instructed to do the following core exercises on a regular basis: sit-ups, the plank, bird-dog and pelvic floor exercises.

The low-to-moderate intensity group exercises at 50% of six- (the first weekly session) and ten repetitions maximum (the second weekly session) corresponding to 12 and 20 repetitions in three sets (reporting 5–7 on the Omni-scale for perceived exertion (39)). Rest periods between sets are two and one minute for the two sessions, respectively. The high intensity group exercises at six- (the first weekly session) and ten repetitions maximum (the second weekly session) corresponding to six and 10 repetitions in three sets (reporting 9–10 on the Omni-scale for perceived exertion (39), with the last set continuing to failure. Rest periods between sets are two and one minute for the two sessions, respectively. Relative exercise intensity is adjusted over the remaining intervention period according to repeated measures of six- and ten repetitions maximum.

### Endurance exercise

The first six weeks after inclusion the participants familiarize themselves with the use of the heart rate monitor and perceived exertion using the Borg-scale [40] for monitoring of exercise intensity and perceived exertion. Four endurance sessions are conducted at the gym and supervised by a coach. Thereafter, the endurance exercise is home-based and followed up by a coach. All participants are instructed to warm-up for approximately 10 min at moderate intensity before each endurance session. The participants are instructed to wear the heart rate monitor during every session and report perceived exertion in a diary. Exercise frequency is recommended to be 2–4 times a week. The low-to-moderate intensity group do continuous-based exercise (running, cycling, walking uphill or any other endurance-based activity) in bouts of at least 10 min at an exercise intensity of 40–50% of the heart rate reserve. The main aim is to reach 150 min of moderate intensity per week. The high intensity group conduct high-intensity interval exercise at an exercise intensity of 80–90% of the heart rate reserve (at the end of the 3rd session) with two minutes exercise (running, cycling, walking uphill or any other endurance-based activity) and two minutes rest between sessions. The participants start with five sessions, increasing to six after the six weeks familiarization period, thereafter adding one session every fourth week until 10 sessions are reached as the maximum, corresponding to 75 min of vigorous intensity per week.

### Behaviour change techniques

Self-regulatory BCTs are provided for the H + BCTs and the LM + BCTs groups. These are strategies to facilitate adherence to the high and low-to-moderate intensity exercise programs, respectively. These support strategies focus on the adherence to the exercise intervention, primarily on the endurance exercise, as it is home-based, and on maintaining exercise according to individual preferences after the completion of the interventions. The BCTs include a) *behavioural goal-setting*, b) short-term *action planning*, c) *self-monitoring,* d) *review of behaviour goals,* e) *problem solving* and *functional behaviour analysis* to identify affect individual-specific determinants of exercise behaviour, and f) long-term *coping planning* to maintain physical exercise by own choice after the intervention is completed. In addition, motivational aspects are explored by initial interviews based on a) previous experiences with physical exercise, b) persons’ outcome expectations for following the exercise intervention as prescribed, c) persons’ anticipated barriers and facilitators following the exercise intervention, and d) self-efficacy to partake in planned weekly exercise. The BCTs are provided according to protocol, but are individually tailored according to each participant’s need and functional behavioural analyses. Each participant meets their coach on a regular basis during the program, with gradually decreasing frequency in order to enhance self-regulation at the end of the intervention. These coaching sessions take place either in connection with the scheduled exercise sessions or via phone. Participants with access to internet can use electronic and easily accessible self-monitoring of exercise behaviour in a web portal developed for the Phys-Can study (described below).

### Education of coaches

Coaches (physiotherapists and personal trainers) have been trained to provide the interventions in the four groups. The education consisted of three common course days for all coaches and three additional days for those providing the BCT conditions with home assignments between course days. The education included lectures and seminars on cancer and cancer treatment, exercise physiology, and repeated practice sessions in the gym according to the intervention protocol on high and low/moderate exercise. Additional lectures and practice on exploration of motivation and use of BCTs were given to coaches providing the BCT conditions.

Written intervention protocols have been developed, specifying all intervention components in the four groups. The coaches are to follow the protocol closely, keep logs of attendance at gym sessions, check heart rate during endurance sessions and monitor any adverse advents related to exercise. Adverse events caused by the exercise are registered in a web-portal (described below). Grade 1 (e.g. muscle strain) means that the participant have to terminate the ongoing specific exercise but can continue with the exercise session. Grade 2 (e.g. fall in blood pressure) means that the participant must terminate the exercise session. Any severe adverse events (e.g. fracture) are reported directly to the PI and managed by healthcare.

To address the coaches’ fidelity to the intervention protocol, research staff visit the gyms repeatedly during the intervention, giving feedback on both physical exercise and use of BCTs. Regular project group meetings are held with all project personnel, where issues about delivering the intervention are discussed and solved. In addition, one coach representative from each study site takes part in monthly Skype meetings where common issues relating to the intervention are discussed and coordinated. Coaches’ use of BCTs is monitored by audio recordings of conversations between coaches and participants, and individual feedback is given by one of the research staff.

### Data monitoring and communication with persons

A web-portal has been designed. The aim of the Phys-Can web portal is to facilitate collection of outcome measures in the multiple study sites and to enable easily accessible electronic self-monitoring of exercise behaviour. The participants sign on to the web portal and complete forms related to outcome assessments and self-monitoring of endurance training included as part of the intervention (those who have no access to internet fill out paper forms). The data base is located at Uppsala University and constructed according to security principles from the Division of Security at Uppsala University. All communication with the data base is encrypted and backups are performed on a regular basis in order to secure data. The web portal automatically sends e-mails or text messages to participants when the scheduled assessment is available, informing them to long on to the portal to complete the questionnaires. Participants also receive reminders by e-mail or text messages. Those who choose to complete paper forms receive the questionnaires and reminders by regular mail. The web portal also notifies research assistants by e-mail when an assessment point is about to open for a specific participant and alerts the research assistants if questionnaires are not completed within a set time frame. This enables precise monitoring of time points for both participants who use the web portal or choose to fill out paper forms. At assessment points when blood samples, fitness tests and recordings of physical activity are included, a research assistant contacts participants by phone to schedule an appointment.

### Enrolment and outcomes (Fig. 2)

**Fig. 2:**
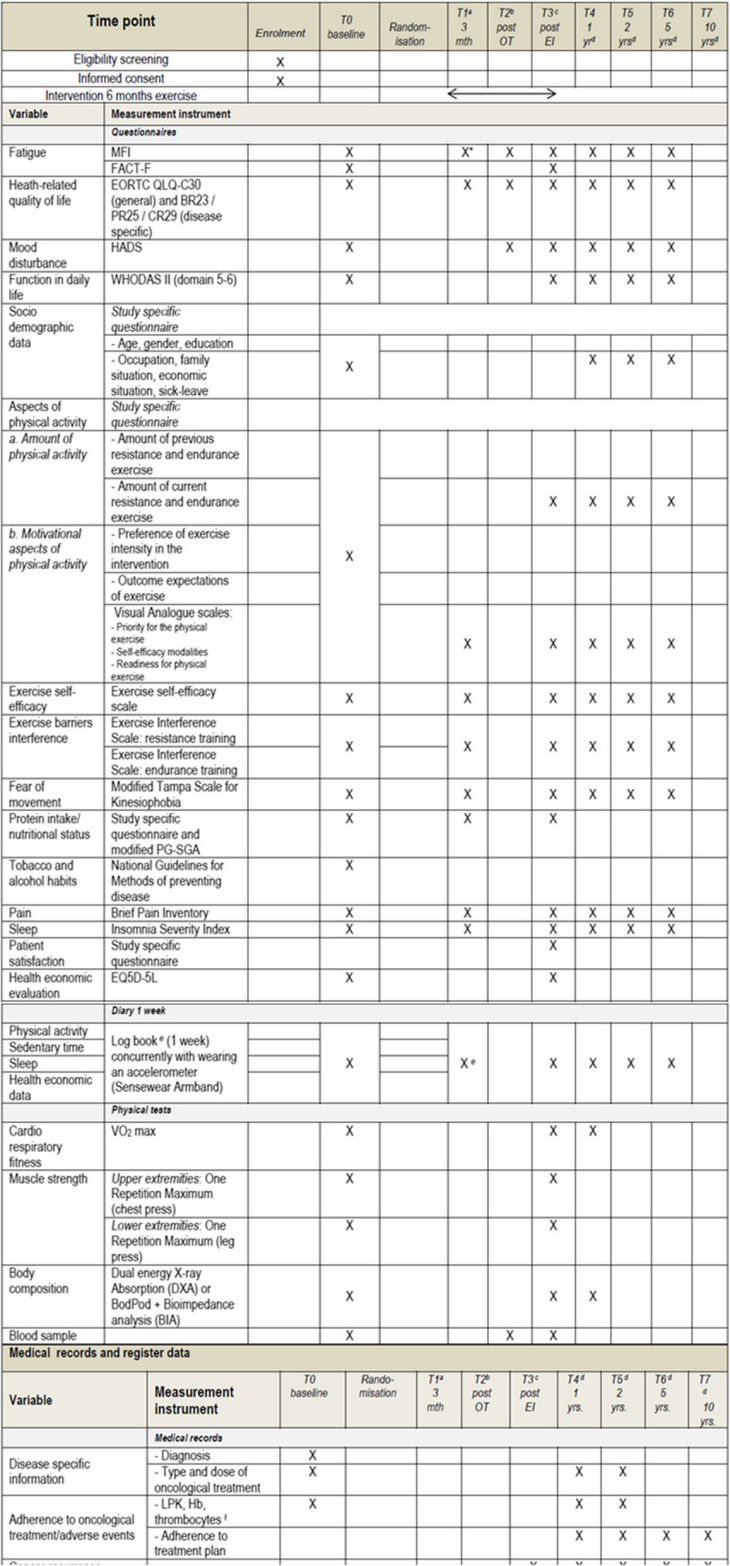
Enrolment and outcome measurements in Phys-Can intervention trial.^*a*^Midway through exercise intervention (3 months), ^*b*^ Directly after end of primary Oncological Treatment (OT), only participants receiving radiation therapy or chemotherapy, ^c^ Directly after end of Exercise Intervention (EI), ^d^ After end of EI, ^e^ Self-reported data on physical activity, sleep onset/end, and health economics are collected via diary during one week concurrently as wearing the Sensewear Armband. At T1, only sleep onset/end and health economics are recorded, ^f^ In participants receiving chemotherapy or radiation therapy, these variables are collected at a regular basis in clinical praxis. *MFI subscale physical fatigue

#### Primary outcome measures

Two Patient reported outcome measures (PROMs) are used to assess CRF: The MFI [41] and the Functional Assessment of Cancer Therapy: Fatigue, FACT-F [42]. The MFI is a 20-item PROM and covers the following dimensions: General Fatigue, Physical Fatigue, Mental Fatigue, Reduced Motivation and Reduced Activity.

#### Secondary outcome measures

The EORTC QLQ-30 [43] and diagnosis-specific modules (QLQ-PR25 for prostate cancer, QLQ-BR25 for breast cancer, QLQ-CR29 for colorectal cancer) are used to assess *HRQoL*.

*Mood* is assessed with Hospital Anxiety and Depressions scale HADS [44] and *Functioning in daily life* with the World Health Organization Disability Assessment Schedule WHODAS II [45].

Readiness to change physical activity behaviour (The Exercise Stage Assessment Instrument (ESAI) [46]), Exercise Barrier Self-Efficacy Scale (ESES) [47, 48], and study specific questions about outcome expectation, progressive goal attainment and perceived behavioural control are used to assess *cognitive-behavioural moderators and mediators*.

*Cardiorespiratory fitness* is measured as maximal oxygen uptake during maximal walking/running until exhaustion on a treadmill using a modified Balke-protocol [49] starting at 4 km/h with an inclination of 2%. The inclination increases with 1% each minute until reaching 12%, from which the speed increases 0.5 km/h per minute until exhaustion. Self-perceived exertion is recorded using a standardized Borg-scale [40]. Oxygen consumption and minute ventilation are measured continuously using an oxygen analyzer. Heart rate is measured using a heart rate monitor. *Maximal* upper and lower extremity *muscle strength* is assessed as one repetition maximum including chest press and seated leg press. Objectively monitored *physical activity* level, *sedentary time* and *sleep* are recorded with SenseWear Armband Mini (BodyMedia Inc., Pittsburgh, PA, USA), also found feasible and valid in cancer persons [50, 51]. The participants wear the monitor for seven consecutive days. The cut off points defining sedentary time and moderate-to-vigorous intensity physical activity are below 1.5 and above 3 metabolic equivalents (METs), respectively. The data from the monitor will be downloaded and analysed with software developed by the manufacturer (Sensewear Professional Research Software Version 8.1, BodyMedia Inc., Pittsburgh, PA, USA).

*Medical and clinical background data* are collected from the records of the Regional Quality Registers and from case records covering treatment administrated, dose intensity, toxicity and adverse events according to NCI-CTC 4.0 and time to cancer recurrence and survival. The Statistics and Result database (STORE) will be used to obtain data on sick days and return to work. The STORE database is managed by the Swedish Social Insurance Agency, and contains information on social insurance for all Swedish residents.

Data from several registries are included and data on persons’ and relatives’ *costs* (time and money) *related to health care utilization* will be collected. Euroqol EQ5D [52] is used to calculate *Quality-adjusted life years* (QALYs).

*Blood samples* are drawn and analysed for the levels of IL-6, IL-8, IL-1β, TNF-α, P-CK-MB, P-CRP, IGF-1, B-HbA1C, P- Cholesterol, P-HDL and P-LDL. Frozen sera are saved in bio-banks for further analyses that can be included later. *Muscle cellular outcomes* will be evaluated on muscle biopsies obtained form *m. vastus lateralis* in a subsample of women with breast cancer. A total of 200 mg tissue will be collected at three time points; before the start of adjuvant chemotherapy, in the middle of treatment, and after the end of treatment. Muscle tissue will be divided into four pieces before further handling and freezing: 1) 20–30 mg in a nice bundle of fibres is secured for later immunohistochemically analyses (fibre area, fibre type, myonuclear number and satellite cells), 2) 50 mg designated for later homogenization and protein analyses (markers of cellular stress (HSPs and inflammatory markers), mitochondrial proteins, regulators of cell size and structural proteins), 3) 30 mg is put in RNA later for later RNA extraction and mRNA analyses, 4) 30 mg is put in RNA later for later morphological analyses on single fibres (nuclear arrangements etc.). Blood and biopsies are stored according to Swedish law in bio-banks (Uppsala IVO ref. nr. 827, Linköping IVO 519, Lund IVO 136).

### Statistical analysis

The data will be analysed according to the intention-to-treat (ITT) principle, i.e. all participants will be analyzed as randomized, regardless of whether they complete the intervention as planned. The primary parameters of interests are the three factorial effects (2 main and one interaction) after completed intervention. Each factorial effect will be estimated taking into account the stratified design following Imbens and Rubin [53]. Thus, we will provide estimates for the overall factorial effects, which are the primary parameters of interest, and also stratum-specific factorial effects which could facilitate external validity. Baseline measurements will be included in the analysis model to increase the precision of the estimates.

Drop-outs and missing values will be handled according to the following procedure. The main analysis will be based on data where missingness is handled using multiple imputations by chained equations. The specification of the multiple imputation model (e.g. choice of auxiliary variables) will be decided after comparing the subject characteristics in those with complete and incomplete data. Under the assumption of missing at random (MAR), an analysis based on multiple imputation conforms with the ITT. Due to the assumption of MAR, which could be a strong assumption to make, factorial effects will also be estimated using complete blocks only. This is a result from the permuted block design which allows for unbiased estimation of the factorial effects using the complete blocks only. Sensitivity analysis will conducted by comparing estimates from the two analyses above with estimates based on complete cases only, a pattern-mixture model, and tipping points analyses [54].

## Discussion

The Phys-Can study aims to determine the effects of low-to-moderate and high intensity physical exercise with or without BCTs on CRF and HRQoL in persons with cancer, both during treatment and in the long-term, post-treatment survivorship period. Additionally, we will investigate the role of inflammation, cytokines and gene expression in the development and maintenance of CRF, as well as the cost-effectiveness of physical exercise programs during cancer treatment.

Systematic reviews underline the potential for physical and psychosocial benefits from rehabilitation programs including physical training [7]. Courneya et al. [10] pointed out in a recent publication the top 10 research questions related to physical training and cancer survivorship and several of them are covered in the present study, e.g. to further investigate the optimal exercise prescription (e.g. intensity), if exercise reduces the risk for cancer recurrence and influences treatment completion rates, the role of behaviour change techniques and variables that may modify/mediate the responses to exercise.

The primary outcome of the present study is CRF since that is one of the most common side effects in persons treated for cancer [7]. Based on data supporting the beneficial effects of physical exercise during oncological treatment, we will perform a large scaled, well-designed study addressing the optimal level and intensity of exercise training to prevent or reduce fatigue and improve HRQoL of persons with cancer and cancer survivors. To also identify potential biological mechanisms and underlying beneficial effects of exercise, blood samples will be analysed, and muscle biopsies obtained from a sub-sample combining an experimental pre-clinical part with an intervention implemented in the oncology clinic.

We will also perform rigorous and maximal testing of physical fitness to tailor the exercise program to the individual participant fitness level as recommended [22]. Carefully monitoring of perceived exertion, heart rate monitors and number of repetitions and loads lifted is important to control and adjust exercise intensity [26]. Furthermore, including BCTs to improve exercise adherence and maintain behavioural changes in the long-term is highlighted as important [26].

The international research group includes experts within oncology, exercise physiology, cell biology, physiotherapy, psychology, cancer rehabilitation, health economy and behavioural medicine, enabling a progressive approach to gain new knowledge. The project will contribute with knowledge also to be used in clinical practice by evaluating high versus low-to-moderate intensity physical exercise, as well as the use of BCTs to adopt and maintain physical activity behaviour. Evaluating the effects of physical exercise as well as identifying moderating and mediating variables on our outcomes CRF and HRQoL can be expected to be beneficial on at least three levels. Individual gains may be improved well-being and quality of life, facilitated return to work, and possibly reduced risk of cancer recurring. This in turn may result in lower burden on the health care system, reduced societal costs positively influencing public health. Implementation of the results into clinical practice will be facilitated by the close collaboration between researchers and clinicians, and the fact that the study intervention is performed in non-clinical settings. The cooperation with public gyms outside healthcare is likely to enable a smooth transition from study setting to maintained exercise by self-management. During the whole process, from planning to implementation of the study, patient representatives are actively involved.

### Methodological discussion

Even though the main aim of the present comparative effectiveness study is to compare different exercise intensities on CRF and HRQoL, the lack of a randomized usual care control group may be a limitation. Due to strong evidence that physical exercise is beneficial for persons during cancer treatment we considered it unethical to randomise persons to an untreated control group that will not be offered exercise. To form a historical cohort, an observational study, was initiated and data collected preceded the randomised study. Persons with the same diagnosis and with the same inclusion/exclusion criteria as in the intervention study were included in the cohort study at all three recruiting centres. The inclusion started in September 2014 and ended immediately prior to start of inclusion to the intervention study. A total of 95 participants were included in the observational study and will be followed for 10 years with same outcomes measures as in the intervention study.

The sample size has been calculated to be 600 persons in total, which is a challenge in a multicentre, complex intervention study. However, a feasibility study (manuscript submitted) preceding the randomized study evaluated the inclusion procedures, settings, intervention components, instruments, and tests.

The lesson learned from the feasibility study has contributed in several ways both to the final planning of the Phys-Can trial. All the physical fitness tests, physical activity monitoring, and conducting exercise of low-to-moderate or high intensity, seem feasible to implement in exercise oncology interventions. In addition, exercise including resistance training with arm movements above the head and repetitive muscle contractions of the arm is feasible and may be safe for patients wearing a peripherally inserted central venous catheter (PICC) (a venous access used for chemotherapy administration is a) due to adjuvant chemotherapy. The results highlighted the need for an enhanced learning and reporting regarding endurance exercise and checking of intensity levels. The results also implied that BCT do not have to target the exercise behavior during supervised sessions; rather, the support should target behaviors pertaining to unsupervised or home-based exercise.

In summary, the Phys-can study will contribute to our understanding of the value of exercise and exercise intensity in preventing CRF and maintaining HRQOL during and after treatment and, potentially, clinical outcomes as well. It will also provide insights into possible biological mechanisms through which exercise affects treatment outcomes. The value of BCTs in terms of adherence to, and maintenances in, exercise behaviour in persons with cancer will be evaluated. Implementation of the results into clinical practice will be facilitated by the close collaboration between researchers and clinicians as well as the facts that the study is performed in non-clinical settings (public gyms) which may create a pathway between hospitals to society.

## Abbreviations

BCT: Behaviour change techniques
CRF: Cancer related fatigue
EORTC QLQ-30: The European organization for research and treatment of cancer quality of life questionnaire
FACT-F: Functional assessments of cancer therapy
HADs: Hospital anxiety and depression scale
HRQoL: Health related quality of life
ITT: Intension to treat
MAR: Missing at random
MCID: Minimal clinically important difference
METs: Metabolic equivalents
MFI: Multidimensional fatigue inventory
MFI-PF: Multidimensional fatigue inventory-physical function
PICC: Peripherally inserted central venous catheter
PROMs: Patient reported outcome measures
QALYs: Quality adjusted life years
WHODAS: World health organization disability assessment schedule
